# A Mysterious Case of Recurrent Confusion: An Unusual Presentation of Congenital Intrahepatic Portosystemic Shunt

**DOI:** 10.7759/cureus.98579

**Published:** 2025-12-06

**Authors:** Fatma Shah, Anesa Noor, Natasha Wahedna, Dipesh Chauhan, Muhammad Shahzad

**Affiliations:** 1 Gastroenterology, Northampton General Hospital, Northampton, GBR; 2 Internal Medicine, Leicester Royal Infirmary, Leicester, GBR; 3 Pharmacy, Northampton General Hospital, Northampton, GBR

**Keywords:** ammonia level, congenital liver diseases, congenital porto-systemic shunt, hepatic encephalopathy, l-ornithine-l-aspartate, porto-systemic shunt

## Abstract

Congenital portosystemic shunts (CPSS) are rare vascular malformations that divert portal blood into the systemic circulation, bypassing hepatic metabolism. Although typically diagnosed in infancy due to associated congenital anomalies or complications such as hepatic encephalopathy, adult presentations are exceptionally uncommon and diagnostically challenging. We report a 71-year-old female patient with recurrent episodes of confusion and drowsiness who was found to have markedly elevated serum ammonia levels despite normal liver function. Extensive investigations excluded infective, metabolic, and structural causes, and a triple-phase liver computed tomography (CT) revealed multiple intrahepatic portosystemic shunts with aneurysmal dilatation, consistent with congenital origin. Due to multiple comorbidities, the patient was deemed unsuitable for shunt closure or liver transplantation but responded well to medical therapy with lactulose, rifaximin, sodium benzoate, glycerol phenylbutyrate, and L-ornithine L-aspartate, with no further episodes of encephalopathy on follow-up. This case underscores the importance of considering CPSS in adults presenting with hyperammonaemia and altered mental status in the absence of hepatic disease. Early recognition and multidisciplinary management are key to preventing recurrence and optimising outcomes.

## Introduction

Congenital portosystemic shunts (CPSS) are rare developmental vascular anomalies that permit portal venous blood to bypass hepatic metabolism and drain directly into the systemic circulation [1]. They may be intrahepatic or extrahepatic and are often identified in infancy or childhood due to their association with other congenital anomalies, such as cardiac malformations or chromosomal syndromes. Adult presentations are exceptionally uncommon and can present a significant diagnostic challenge, particularly in the absence of overt hepatic disease.

The clinical spectrum of CPSS varies widely from asymptomatic cases to severe complications such as hyperammonaemic encephalopathy, pulmonary hypertension, and hepatic nodular lesions [2]. Diagnosis typically requires high-resolution cross-sectional imaging, including computed tomography (CT) or MRI with intravenous contrast.

Management depends on the severity of symptoms, anatomical type of shunt, and hepatic function. Conservative approaches involve ammonia-lowering therapies (lactulose, rifaximin, sodium benzoate, or L-ornithine L-aspartate (LOLA)) and dietary protein modification. In refractory or anatomically suitable cases, interventional shunt closure via embolisation may be attempted, whereas liver transplantation remains an option in cases that are not amenable to closure. This report describes a rare case of congenital intrahepatic portosystemic shunts (CIPSS) presenting in late adulthood, managed successfully with medical therapy alone.

## Case presentation

A 71-year-old female patient presented with an acute onset of confusion and drowsiness. On assessment, she was haemodynamically stable with blood pressure of 118/75 mmHg, heart rate of 100 beats per minute, and oxygen saturations of 99% on room air and was afebrile. She was, however, unconscious with a Glasgow Coma Scale (GCS) of 6/10 (E1V2M3). She was, therefore, intubated in the emergency department. The rest of her physical examination was normal.

Her past medical history included systemic hypertension, paroxysmal atrial fibrillation, and permanent pacemaker insertion due to symptomatic sinus node dysfunction. The patient previously had severe symptomatic right-sided heart failure and torrential tricuspid regurgitation, which was managed with tricuspid edge-to-edge repair (TEER). During the same procedure, she also underwent percutaneous left atrial appendage occlusion due to intolerance to anticoagulation, which caused recurrent anaemia requiring multiple transfusions.

Prior to admission, the patient had multiple hospital attendances in the preceding 24 months with short-lived intermittent episodes of acute agitation and confusion, all of which self-resolved within a few days. Some episodes were felt to be delirium in the context of simple lower urinary tract infections. Additionally, she was seen in the memory clinic and by the psychiatry team; no cognitive or psychiatric cause for her symptoms was identified.

Investigations

Routine initial investigations were overall unremarkable. However, liver function tests were mildly deranged, and platelets were borderline low (Table 1). Ammonia levels were noted to be raised at 125 μmol/L (normal range 11-51).

**Table 1:** Blood results CRP: C-reactive protein; eGFR: estimated glomerular filtration rate; GGT: gamma glutamyl transferase; TSH: thyroid-stimulating hormone; free T4: free thyroxine; DsDNA Ab: anti-double-stranded deoxyribonucleic acid antibody; ANA: antinuclear antibody

Investigations	Results	Normal range
CRP	2 mg/L	0-5
Urea	5 mmol/L	2.5-7.8
eGFR	79 mL/min	
Sodium	145 mmol/L	133-146
Potassium	4.3 mmol/L	3.5-5.3
Creatinine	67 μmol/L	45-84
Bilirubin	31 μmol/L	0-21
Alkaline phosphatase	134 IU/L	30-130
Alanine transaminase	24 IU/L	5-33
GGT	43 IU/L	0-38
Corrected calcium	2.47 mmol/L	2.2-2.6
Troponin	<13 ng/L	
Haemoglobin	122 g/L	120-150
White cell count	4.6 × 10^9^/L	4-10
Platelets	136 × 10^9^/L	150-400
Neutrophils	3.95 × 10^9^/L	1.8-7.4
TSH	4.3 mU/L	0.27-4.2
Free T4	16.2 pmol/L	12-22
DsDNA Ab	26.7 IU/mL	0-27
ANA	Centromeric pattern	
Free carnitine	20 μmol/L	15-53

A CT scan of the head showed no parenchymal haemorrhage or extra-axial collection. The patient also underwent a CT thorax, abdomen, and pelvis, which showed mild bilateral pleural effusions with passive atelectasis. Liver morphology and size were normal.

Lumbar puncture was performed; cerebrospinal fluid (CSF) demonstrated normal white cell count and glucose. CSF protein was elevated at 0.98 g/L (0/15-0.45). Table 2 details the CSF antibodies checked. Blood, urine, and CSF cultures, fungal markers, hepatitis B and C virus, human immunodeficiency virus (HIV), herpes simplex virus, enterovirus, cytomegalovirus, chlamydia, and syphilis serology were all negative.

**Table 2:** CSF investigations GABA-B receptor: gamma-aminobutyric acid type B receptor; CSF: cerebrospinal fluid

CSF antibodies	Results
N-Methyl-D-aspartate (NMDA)	Negative
Voltage-gated potassium channel	Negative
Anti-contactin-associated protein 2 (CASPR)	Negative
Anti-leucine-rich glioma inactivated 1 (LGI1)	Negative
Anti-AMPA-1 (α-amino-3-hydroxy-5-methyl-4-isoxazolepropionic acid receptor)	Negative
Anti-AMPA-2	Negative
GABA-B receptor	Negative

A transthoracic echocardiogram (TTE) followed by a transoesophageal echocardiogram (TOE) ruled out infective endocarditis. An MRI head could not be performed due to her pacemaker incompatibility. EEG showed diffuse slow wave activity, suggesting encephalopathy of unclear origin. Autoimmune screen was negative, and anti-nuclear antibody (ANA) showed a centromeric pattern.

Differential diagnosis

The patient was initially treated empirically with intravenous antibiotics and antivirals to cover possible infective encephalitis as a cause for her presentation; however, this treatment was discontinued after CSF PCR and culture results excluded viral or bacterial infection. The patient’s elevated ammonia was initially thought to be due to a urea cycle disorder because of the normal appearance of the liver on imaging. The case was discussed with the national adult inherited metabolic disorder team. Plasma organic acids showed mildly raised methionine, tyrosine, glutathione, and phenylalanine, whilst urine organic acids were normal. This made a diagnosis of an inherited metabolic disorder less likely.

**Figure 1:**
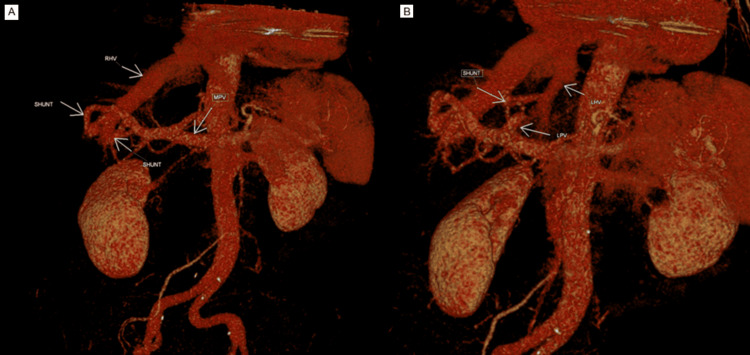
(A, B) CT shows the presence of shunts in both lobes of the liver RHV: right hepatic vein; MPV: main portal vein; LHV: left hepatic vein; LPV: left portal vein; CT: computed tomography

**Figure 2:**
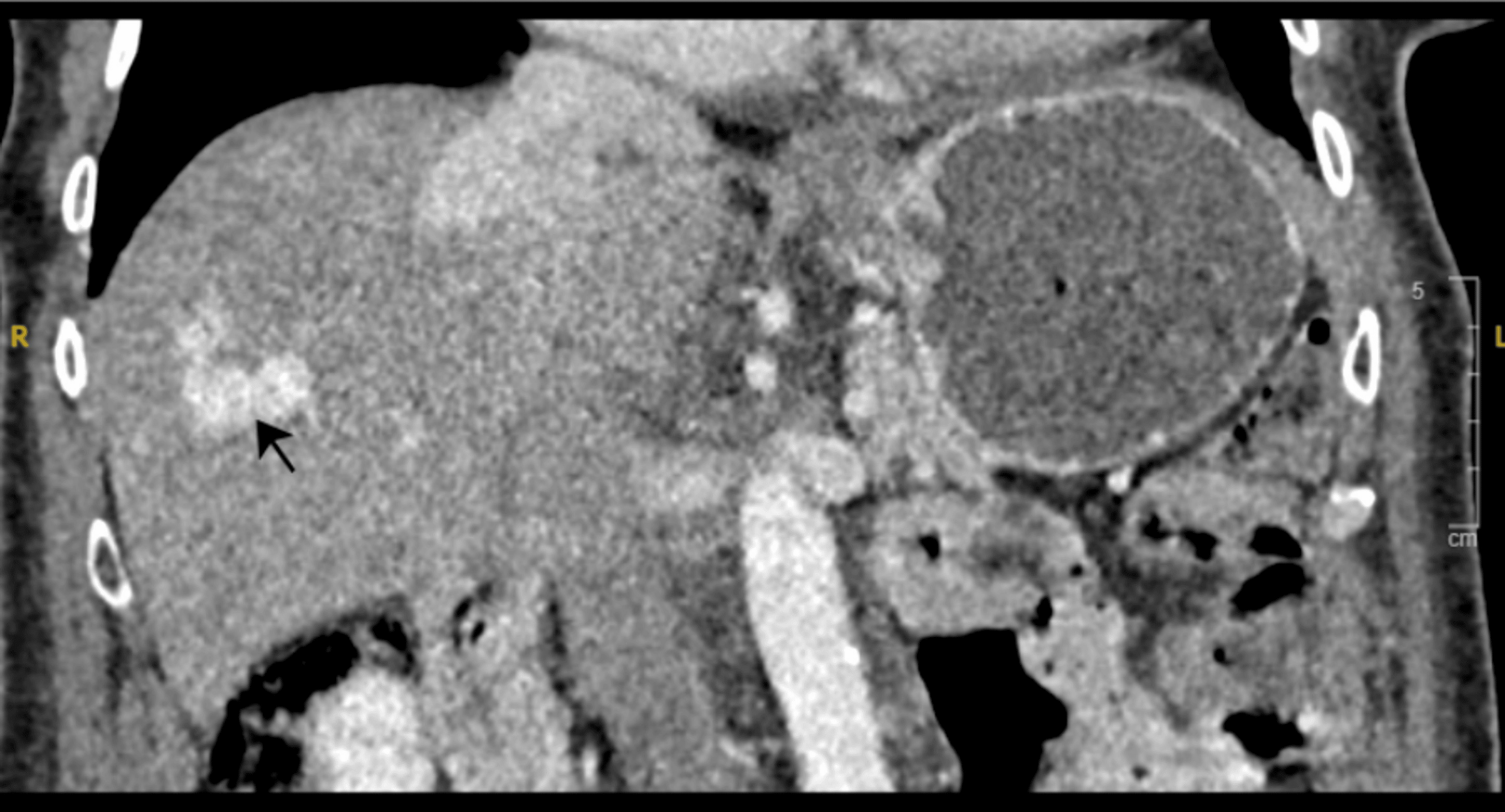
CT scan showing aneurysmal enlargement of shunts CT: computed tomography

Liver dysfunction or portosystemic shunting was suspected as the more likely cause for the raised ammonia level. The case was discussed with the gastroenterology team, who advised performing a triple-phase liver CT. This showed multiple intrahepatic communications/shunts between the peripheral portal and hepatic veins (Figure 1), with concerning aneurysmal enlargement of a few of these channels (Figure 2). The shunts were present in both lobes of the liver, with the right shunt being larger than the left. CT images were reviewed retrospectively, and they confirmed the presence of shunts as early as 2013.

Treatment

The raised ammonia levels were initially refractory to regular high-dose lactulose despite daily bowel movements. Therefore, she required continuous veno-venous haemofiltration (CVVH) for 72 hours during her stay on the intensive care unit, leading to an improvement in her ammonia and conscious level. Subsequently, she was commenced on additional ammonia-lowering therapy, including rifaximin, sodium benzoate, and later glycerol phenylbutyrate.

Her case was discussed in the regional hepatology multidisciplinary team (MDT) meeting, and the consensus was that these intrahepatic/portosystemic shunts were likely congenital. Given the patient's comorbidities, she was deemed unsuitable for liver transplantation, and the plan was to continue medical management. It was also felt that the closure of the shunts in her case would be high risk with uncertain benefit. LOLA was added to her other therapies on the advice of the MDT to improve ammonia control.

Outcome and follow-up

The patient was discharged after her confusion improved. She has not had any further admissions with delirium since commencing maintenance treatment with lactulose, rifaximin, glycerol phenylbutyrate, and LOLA.

## Discussion

CPSS are rare occurrences with an incidence of about 1:30,000 live births [1]. They are divided into two types: intrahepatic and extrahepatic shunts. Extrahepatic portosystemic shunts (EPSS) are then classified into type 1 ‘Abernethy malformations’, in which there is a complete absence of portal flow to the liver, and type 2, where there is only a partial flow into systemic circulation [3]. Hepatic encephalopathy (HE) is common in both types [4].

Intrahepatic portosystemic shunts (IPSS) are classified into four types as per Park et al.’s criteria proposed in 1990: a single vessel communication, which can be either between a main branch of the portal vein and inferior vena cava (IVC) (type 1), peripheral location in one segment (type 2), or through an aneurysm (type 3), and multiple small communications distributed diffusely in both lobes (type 4) [5]. Some newer classifications have been proposed depending on severity and response to treatment (Kanazawa et al.), shunt origin (Lautz et al.), and shunt drainage (Kobayashi et al.) [6-8].

CPSS are usually diagnosed at birth or infancy, as they are linked with other congenital disorders, especially cardiovascular, such as ventricular and atrial septal defects, patent foramen ovale, patent ductus arteriosus, and tetralogy of Fallot [2]. Important complications can include pulmonary vascular hypertension and hepatopulmonary syndrome. Additionally, CPSS can be linked to the formation of hepatic nodules that may have a malignant potential, highlighting the importance of regular surveillance.

CPSS are very rarely diagnosed in adults. A case of type 1 IPSS with recurrent HE was described in a 20-year-old with mildly deranged liver functions [9]. Similarly, an elderly patient with neurological symptoms, including left arm paresis and rapidly declining cognition, was described to have IPSS [10]. These cases highlight that there should be a high suspicion index for CIPSS in the absence of other pathologies and hyperammonaemia in the absence of liver cirrhosis.

Conservative management for HE includes avoiding precipitating factors such as constipation, alcohol, infection, and gastrointestinal bleeding. Lactulose and rifaximin are commonly used to prevent and treat HE. Sodium benzoate is approved for use in patients with hyperammonaemia secondary to urea cycle disorders. Additionally, trials have found that it also significantly improves ammonia concentrations in patients with liver disease [11]. However, sodium benzoate can only be administered intravenously; therefore, sodium phenylbutyrate and its prodrug, glycerol phenylbutyrate, can both be administered orally as an alternative. Both options have been observed to significantly reduce HE in cirrhotic patients compared to control groups [12,13]. LOLA is an off-licence treatment in the United Kingdom, which reduces serum ammonia concentration. It acts as a substrate in the urea cycle, thereby enhancing ammonia detoxification by increasing urea synthesis, and as a substrate in glutamine synthesis, in which it is converted to glutamate. This increases the reversible detoxification of ammonia systemically via glutamine syntheses in the brain and muscles [14]. A meta-analysis looking into the efficacy of LOLA in preventing and treating overt HE in the context of cirrhosis or post-TIPSS showed a reduction of ammonia levels with LOLA use [15].

Closure of shunts is recommended in patients with systemic complications, if feasible. However, this intervention presents the risk of secondary portal hypertension. Additionally, it is contraindicated in patients with severe liver disease or the presence of hepatocellular carcinoma (especially large or multifocal lesions), necessitating assessment of risk versus benefit on a case-by-case basis [16]. Closure is not feasible in patients with type 1 CPSS; patients with refractory encephalopathy and/or hepatopulmonary syndrome should be considered for liver transplant, and several cases have reported resolution of associated complications thereafter [17].

## Conclusions

This case highlights a rare presentation of CIPSS diagnosed in late adulthood, manifesting as recurrent episodes of encephalopathy with hyperammonaemia in the absence of liver disease. The diagnosis required a high index of suspicion and a comprehensive multidisciplinary evaluation to exclude more common causes. Recognition of such anomalies is crucial, as timely identification and initiation of targeted ammonia-lowering therapies can significantly improve outcomes and quality of life. Although definitive interventional procedures may be contraindicated in patients with multiple comorbidities, optimal medical management can achieve sustained neurological stability. This case underlines the importance of considering CIPSS in unexplained encephalopathy with normal hepatic function and reinforces the role of collaborative care in managing complex metabolic and vascular disorders.
